# The Impact of Technological Progress in the Energy Sector on Carbon Emissions: An Empirical Analysis from China

**DOI:** 10.3390/ijerph14121505

**Published:** 2017-12-04

**Authors:** Lei Jin, Keran Duan, Chunming Shi, Xianwei Ju

**Affiliations:** 1 School of Business Administration, China University of Petroleum, Beijing 11414, China; jinlei@cup.edu.cn (L.J.); cupjuxianwei@163.com (X.J.); 2 Lazaridis School of Business and Economics, Wilfrid Laurier University, Waterloo, ON N2L 3C5, Canada; Cshi@wlu.ca

**Keywords:** carbon dioxide emissions, technological progress, R & D, energy efficiency, climate change

## Abstract

This paper investigates the relationship between technological progress in the energy sector and carbon emissions based on the Environment Kuznets Curve (EKC) and data from China during the period of 1995–2012. Our study confirms that the situation in China conforms to the EKC hypothesis and presents the inverted U-curve relationship between per capita income and carbon emissions. Furthermore, the inflection point will be reached in at least five years. Then, we use research and development (R & D) investment in the energy industry as the quantitative indicator of its technological progress to test its impact on carbon emissions. Our results show that technological progress in the energy sector contributes to a reduction in carbon emissions with hysteresis. Furthermore, our results show that energy efficiency improvements are also helpful in reducing carbon emissions. However, climate policy and change in industrial structure increase carbon emissions to some extent. Our conclusion demonstrates that currently, China is not achieving economic growth and pollution reduction simultaneously. To further achieve the goal of carbon reduction, the government should increase investment in the energy industry research and improve energy efficiency.

## 1. Introduction

Climate change has come under intense scrutiny, and carbon emissions are considered to be one of the culprits that cause it (Intergovernmental Panel on Climate Change (IPCC) 1997). With a large population and a vast territory, China has huge carbon emissions. China surpassed the United States to become the world’s largest carbon producer in 2006 (Milieu en Natuur Planbureau (MNP) (Netherlands Environmental Assessment Agency)). As a result, the Chinese government is under great pressure to reduce carbon emissions. In 1998, China signed the Kyoto Protocol, which is the first document restricting greenhouse gas emissions in the history of mankind, and approved the protocol in 2002. At the Copenhagen World Climate Conference in 2009, China also made a commitment to achieve a series of targets, including that its proportion of renewable energy use will achieve 16% in the energy structure in 2020.

Technological progress has received more and more attention as a key factor that affects carbon emissions. However, most of the previous scholars have been concerned about overall social technological progress [1,2,3] and not about the impact of technological progress in a specific sector on carbon emissions. There is no doubt that energy consumption is the most direct source of carbon emissions [4], so technological progress in the energy sector can more directly affect carbon emissions. Therefore, this paper will focus on the impact of the energy sector’s technological progress on carbon emissions. Through the analysis of the above impact, and compared with the studies which are only concerned about the impact of overall technological progress on carbon reduction, we will put forward more targeted carbon reduction policy recommendations. The Environmental Kuznets Curve (EKC) can help us examine the effects of the energy sector’s technological progress on carbon emissions.

**Figure 1:**
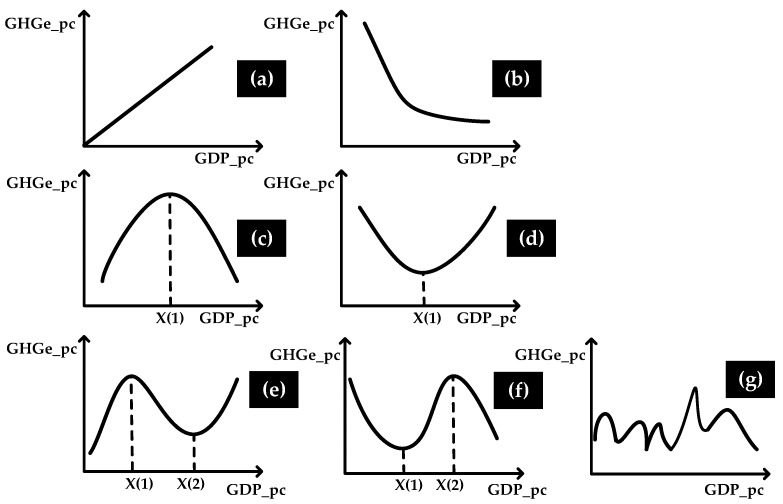
Possible links between environmental quality and GDP per capita. (**a**) If *β*1 > 0, *β*2 = *β*3 = 0, there is an increasing monotonic relationship, such that high levels of income are associated with high levels of pollution; (**b**) If *β*1 < 0, *β*2 = *β*3 = 0, there is a decreasing monotonic relationship, such that high levels of income are associated with decreasing levels of pollution; (**c**) If *β*1 > 0, *β*2 < 0, and *β*3 = 0, a quadratic relationship in an inverted U-shaped pattern indicates that high levels of income are associated with decreasing levels of pollution, beyond a certain level of income; (**d**) If *β*1 < 0, *β*2 > 0, and *β*3 = 0, there is a quadratic relationship in a U-shaped pattern, in direct contrast with the Environmental Kuznets Curve (EKC); (**e**) If *β*1 > 0, *β*2 < 0, and *β*3 > 0, a cubic polynomial reveals an N shape, such that the inverted U-shaped pattern occurs up to a certain point, after which pollution increases again; (**f**) If *β*1 < 0, *β*2 > 0, and *β*3 < 0, we have a cubic polynomial in an inverted N shape; (**g**) If *β*1 = *β*2 = *β*3 = 0, flat behavior indicates that emissions are not influenced by the level of income [8].

The EKC is used to describe the relationship between economic development and the environment. How can we protect the environment from damage while maintaining economic development? Will economic development increase carbon emissions? The answer seems to be yes, but Grossman and Krueger [5,6] have argued that extensive economic growth at the initial stage does lead to environmental pollution. As technology progresses, environmental regulations will become more stringent, pollutant emissions will gradually decline, and ultimately the final pollutant emissions and economic development will show an inverted U-shaped relationship. This relationship is called the EKC. Moreover, the EKC has emerged in a variety of shapes as shown in Figure 1. In theory, the EKC can be decomposed into a scale effect, a structural effect, and a technique effect [7,8]. The scale effect asserts that even if the structure of an economy and technology do not change, economic expansion will bring more pollution. The structural effect means that a country’s economic structure will also change with economic growth. For example, in the process of turning to light industry from agriculture, and then turning to high pollution, high-emission heavy chemical industry, and gradually to a tertiary industry, the emissions continue to change. The technique effect means that technological progress has an effect on the environment. We should add variables to the EKC basic model based on these effects [9].

The research in this paper will be based on the EKC. On the basis of the endogenous model of economic growth and environmental pollution, according to the technical effect of the EKC, this paper examines whether technological progress in the energy sector really plays a role in decreasing emissions and the subsequent slowing of environmental deterioration in China during 1983–2014. Then, we also introduce the influence factors of energy efficiency, government regulation, and industrial structure, and examine their effects on carbon emissions [7,9,10]. The main contribution of this paper is to examine the impact of technological progress in the energy industry on carbon emissions. We believe that technological progress in the energy sector is more relevant than overall technological progress.

The rest of the paper is organized as follows: Section 2 presents a literature review; Section 3 describes the model and data; and Section 4 presents the empirical results in the paper and their implications. Section 5 concludes the paper.

## 2. Literature Review

In recent years, a few scholars have used the EKC to study the relationship between pollutant emissions and economic development. Since the EKC hypothesis was proposed in 1991, various pollutants have been studied based on it. Earlier research focuses on sulfur dioxide and water pollution [11,12,13]. Since the Kyoto Protocol was signed, many scholars have studied the relationship between carbon emissions and economic growth based on the EKC hypothesis. These studies are different in terms of estimation methods, periods, samples, and measurement variables. Additionally, there are inconsistencies about the validity of the EKC hypothesis and the shape of the EKC. We select several recent representative studies, which are presented in Table 1. The first six studies’ dependent variables are other pollutants’ emissions [10,14,15,16,17,18]. Other papers use carbon emissions as the dependent variable [4,19,20,21,22,23,24,25,26,27,28,29,30,31,32].

Comparing the existing studies, we find that when the dependent variable is carbon emissions, and if the samples are from developed countries (because most of the Organisation for Economic Co-operation and Development (OECD) member countries are developed countries, so members of the OECD are seen as developed country samples), the validity of the EKC hypothesis is higher [4,25,26,32]. However, if the samples are from developing countries, the validity of the EKC hypothesis is lower [20,21,22,23,24,27,28,29,30]. There are two possible explanations for this result. First, most of the developing countries do not currently reach the inflection point in the EKC. In other words, these countries are still in the stage where economic growth is synchronized with a carbon emission increase. Second, pollutant emissions continue to decline with national economic development, so the inflection point is not reached. However, the second explanation is not very likely [33]. Therefore, development levels and development paths still differ greatly between developing countries and developed countries. As a rapid developing country, it remains a question whether China is consistent with the EKC hypothesis or not [22,24,27,29]. This study aims to answer this question.

Moreover, some research has investigated the impact of technological progress on carbon emissions based on the EKC. In these papers, the methods used to measure technological progress and their results are different. For example, the number of patents represents the ability of a country to innovate to a certain extent [34], so it can be a measure of technological progress. It has a negative but insignificant relationship with environmental pollution in Malaysia [20]. Some researchers argue that trade openness is an important guarantee for sustained innovation and long-term economic growth, so trade openness can be an indicator of technological progress. Related results show that technological innovation is insignificant in helping reduce carbon emissions [35]. Total factor production (TFP) refers to the combined effect of institutional innovation, technological innovation, industrial structure adjustment, and resource optimization allocation, including labor and capital [36]. It is a powerful means of reducing carbon dioxide emissions in China when it is used as a measure of technological progress [37]. However, more scholars assume that research and development (R & D) investment is a direct cause of technological progress [10,38,39], and this assumption is supported by ‘new growth theory’ or ‘induced technological change’ [40]. However, its impact on carbon emissions is uncertain. Thus, this article uses R & D to represent technological progress and explore its impact on carbon emissions. On the one hand, R & D investment does not immediately translate into innovation and new technologies. As the translation takes time, it contributes to hysteresis. On the other hand, the adoption of new technologies also takes some time. This is true as people tend to stick to old technologies for reasons such as habits and cost savings. Further, scholars have confirmed that the impact of technological progress on carbon emissions has long- and short-term [36] differences. Therefore, this paper takes hysteresis into account when developing the models.

## 3. Model and Data

The initial model of the EKC is shown below [41]:

(1) $$Y_{it}=G_{it}\beta_{1}+G_{it}^{2}\beta_{2}+G_{it}^{3}\beta_{3}+{\bar{G}}_{it-}\beta_{4}+\bar{G_{it-}^{2}}\beta_{5}+\bar{G_{it-}^{3}}\beta_{6}+X_{it}^{\prime }\beta_{7}+\varepsilon_{it}$$

where $Y_{it}$ is a measure of pollution in station i in year t, $G_{it}$ is per capital GDP in station i in year t, ${\bar{G}}_{it-}$ is the average per capital GDP over the prior three years, $X_{it}^{\prime }$ is a vector of other covariates, $\varepsilon_{it}$ is an error term, and β×s are parameters that need to be estimated.

Scholars continually changed the model to meet the requirements of studies until Balsalobre [8] summed up a more complete framework, in which the different shapes correspond to the different EKCs. This general theoretical framework allows us to identify different scenarios (Figure 1) about the relationship between economic growth and environmental quality. The model is as follow:

(2) $$GHG_{pc_{it}}=a_{i}+\beta_{1}GDP_{pc_{it}}+\beta_{2}GDP_{pc_{it}}{}^{2}+\beta_{3}GDP_{pc_{it}}{}^{3}+\beta_{4}Z_{it}+e_{it}$$

where $GHG_{pc_{it}}$ is the index of emissions of greenhouse gases, $GDP_{pc_{it}}$ is the per capita income level, and $Z_{it}$ represents other influences on environmental quality. The a coefficient includes the average environmental quality when income has no special relevance for environmental concerns, the β×s coefficients represent the relative importance of variables, and $e_{it}$ is the error term distributed as a normal of 0 average and constant variance. The sub index i indicates the country or region, and t indicates the time.

### 3.1. Models

First, based on the existing EKC model, we want to identify the relationship between economic growth and environment quality, and Model (3) is considered to be the basic model, which is simpler than the Balsalobre model. We want to identify the basic shape of the EKC, so it can lead to the problem simplified so as to consider only the relationship between the economy and carbon emissions without taking into account the impact of other factors.

(3) $$CO_{2}{}_{pc_{t}}=a+\beta_{1}GDP_{pc_{t}}+\beta_{2}GDP_{pc_{t}}{}^{2}+\beta_{3}GDP_{pc_{t}}{}^{3}+\varepsilon_{t}$$

After proposing the Model (3), we consider that if the whole model or one coefficient cannot pass the test, or the cubic EKC does not conform to the situation in this study, the robustness of the model will need to be strengthened. To ensure robustness, we propose the Model (4). On the basis of the Model (3), we remove the cube of GDP, that is, the Model (4) becomes more simplified as:

(4) $$CO_{2}{}_{pc_{t}}=a+\beta_{1}GDP_{pc_{t}}+\beta_{2}GDP_{pc_{t}}{}^{2}+\varepsilon_{t}$$

Based on the estimation of the Models (3) and (4), we can determine the basic shape of the EKC in this paper and predict whether there is an inflection point in the curve. After ordinary least squares (OLS) regression, we find that Model (2) is more consistent with this study.

Second, after determining the quadratic relationship between carbon emissions and per capita income, the impact of other variables on carbon emissions should be investigated. Although we are studying the impact of technological progress on carbon emissions, because of the scale effect and structure effect of the EKC, other factors cannot be ignored. We add energy efficiency (EE), climate policy (POL), industrial structure (IND), and R & D in the energy sector (ER & D) into Model (4) to form Model (5). $EE_{t}\times GDP_{pc_{t}}$, $POL_{t}\times GDP_{pc_{t}}$, and $IND_{t}\times GDP_{pc_{t}}$ indicate the dampening effect exerted by GDP on energy efficiency, climate policy, and industrial structure, and we estimate the impact of per capita GDP on them by incorporating the above three variables to pick the dampening effect [42]. In short, energy efficiency and GDP can influence each other, $EE_{t}\times GDP_{pc_{t}}$ are interaction terms that capture the interaction between $EE_{t}$ and $GDP_{pc_{t}}$, and the other two are the same. They alter the magnitude or direction of the relationship between the independent variable and the response variable, by amplifying or even inverting their causal effect [10].

(5) $$\begin{array}{c} CO_{2}{}_{pc_{t}}=a+\beta_{1}GDP_{pc_{t}}+\beta_{2}GDP_{pc_{t}}{}^{2}+\beta_{3}EE_{t}+\beta_{4}EE_{t}\times GDP_{pc_{t}}+\beta_{5}POL_{t}+\beta_{6}POL_{t}\\ \times GDP_{pc_{t}}+\beta_{7}IND_{t}+\beta_{8}IND_{t}\times GDP_{pc_{t}}+\beta_{9}ZDL_{t}+\varepsilon_{t} \end{array}$$

where

$$ZDL_{t}=\left[\sum_{j=0}^{s/2}\left(j+1\right)+\sum_{j=s/2+1}^{s=4}\left(s-j+1\right)\right]ER\&D_{pc_{t-j}}$$

In this paper:

$$ZDL_{t}=\left(1\times ER\&D_{pc_{t}}\right)+\left(2\times ER\&D_{pc_{t-1}}\right)+\left(3\times ER\&D_{pc_{t-2}}\right)+\left(2\times ER\&D_{pc_{t-3}}\right)+\left(1\times ER\&D_{pc_{t-4}}\right)$$

This method makes ER & D appear as a dynamic variable of a finite V-lag distribution structure of order 4. It includes that innovation accumulates over time and also fully demonstrates the hysteresis of the technological progress effect. According to Álvarez-Herránz [10], when other variables have an impact on greenhouse gas emissions, the influence intensity grows until it reaches a maximum in the *j* = 2 value, and then the intensity starts to decline. Carbon dioxide is a kind of greenhouse gas, so the above condition is applicable in this research.

(6) $$CO_{2}{}_{pc_{t}}=a+\beta_{1}GDP_{pc_{t}}+\beta_{2}GDP_{pc_{t}}{}^{2}+\beta_{3}POL_{t}+\beta_{4}POL_{t}\times GDP_{pc_{t}}+\beta_{5}IND_{t}+\beta_{6}IND_{t}\times GDP_{pc_{t}}+\beta_{7}ZDL_{t}+\varepsilon_{t}$$

(7) $$CO_{2}{}_{pc_{t}}=a+\beta_{1}GDP_{pc_{t}}+\beta_{2}GDP_{pc_{t}}{}^{2}+\beta_{3}EE_{t}+\beta_{4}EE_{t}\times GDP_{pc_{t}}+\beta_{5}IND_{t}+\beta_{6}IND_{t}\times GDP_{pc_{t}}+\beta_{7}ZDL_{t}+\varepsilon_{t}$$

(8) $$CO_{2}{}_{pc_{t}}=a+\beta_{1}GDP_{pc_{t}}+\beta_{2}GDP_{pc_{t}}{}^{2}+\beta_{3}EE_{t}+\beta_{4}EE_{t}\times GDP_{pc_{t}}+\beta_{5}POL_{t}+\beta_{6}POL_{t}\times GDP_{pc_{t}}+\;\beta_{7}ZDL_{t}+\varepsilon_{t}$$

(9) $$CO_{2}{}_{pc_{t}}=a+\beta_{1}GDP_{pc_{t}}+\beta_{2}GDP_{pc_{t}}{}^{2}+\beta_{3}EE_{t}+\beta_{4}EE_{t}\times GDP_{pc_{t}}+\beta_{5}POL_{t}+\beta_{6}POL_{t}\times GDP_{pc_{t}}+\beta_{7}IND_{t}+\beta_{8}IND_{t}\times GDP_{pc_{t}}+\varepsilon_{t}$$

In order to more closely examine the impact of each variable on carbon emissions and avoid multicollinearity, on the basis of Model (5), Model (6) omits $EE_{t}$ and $EE_{t}\times GDP_{pc_{t}}$, Model (7) omits $POL_{t}$ and $POL_{t}\times GDP_{pc_{t}}$, Model (8) omits $IND_{t}$ and $IND_{t}\times GDP_{pc_{t}}$, and Model (9) omits $ZDL_{t}$.

### 3.2. Data

Annual data from China covering the period 1983–2014 is used for the study. Per capita carbon emissions in kg are obtained from OECD Data. Energy efficiency is the ratio of per capita GDP to per capita energy consumption; climate policy is a variable that differentiates before and after 2002 (in which year China approved the Kyoto Protocol): it is assigned a numeric value of 0 before 2002, while after 2002 its value is one; the industrial structure is expressed as the proportion of the tertiary industry.

ER & D is the sum of the R & D of the coal mining and washing industry, the oil and gas extraction industry, oil processing, coking and nuclear fuel processing, electricity, heat production and supply, and gas production and supply. All of these industries are directly related to energy.

Per capita GDP, energy consumption, the proportion of tertiary industry, and R & D are obtained from the China Energy Statistical Yearbook and the China Statistical Yearbook. Statistical descriptions of all of the variables in Model (5) are shown in Table 2.

## 4. Results

### 4.1. Relationship between Economic Growth and Carbon Emissions

The empirical results for testing the relationship between economic growth and carbon emissions are shown in Table 3. We can see that although the value of R^2^ is high, only the coefficient of $GDP_{pc_{t}}$ can pass the *T* test. So we think that Model (3) does not fit with the data from China. In comparison, all explanatory variables can pass the test at a 1% significance level in Model (4), and the value of R^2^ is greater than 0.99, which indicates that the overall fitting degree is good. The coefficient of $GDP_{pc_{t}}$ is greater than 0, and the coefficient of its square is less than 0. From Figure 1, an inverted “U” curve is obtained. Through calculation, when per capita GDP reaches 64,797.42038 yuan, the inflection point of the EKC will appear. China’s per capita GDP was 49,992 yuan in 2015, and the average growth rate of per capita GDP in the past five years is 10% and it has a clear trend of gradual slowdown. Therefore, it is possible to reach the inflection point of the EKC of carbon emissions in at least five years. In other words, although we prove that China’s development and carbon emissions conform to the EKC hypothesis, the inflection point has not appeared until now. Hence, we are still in the situation of synchronization of development and pollution, and this situation will remain for at least five more years. So, as for environmental governance, especially carbon emissions reduction, the Chinese government still needs to strengthen its control or provide an improvement in methods.

### 4.2. Relationship between Other Factors and Carbon Emissions

Table 4 shows the result of Models (5)–(9) which mainly examine the relationship between technological progress and carbon emissions, but the effects of energy efficiency, government regulation, and industrial structure on carbon emissions are also calculated. We eliminate one or two explanatory variables from Model (5) to respectively construct Models (6)–(9). Next, we discuss the effects of the different factors.

In this paper, increased energy efficiency means that the energy consumption of per unit GDP is reduced. From the regression results of Model (5) and Models (7)–(9), we can see that energy efficiency is negatively correlated with CO_2_ emissions, that is, increased energy efficiency can reduce carbon dioxide emissions. This result is consistent with most previous studies, and it demonstrates that it is feasible to reduce carbon emissions by increasing energy efficiency. The main reason is that the improvement of energy efficiency brings the effective utilization of energy. The energy industry’s technological progress will help the development and utilization of new energy. Additionally, the use of renewable energy or clean energy will greatly reduce carbon emissions [43,44,45], which will further help solve the problem of pollution.

The regression results of Models (5)–(7) and Model (9) tell us that climate policy and CO_2_ emissions are positively correlated, that is, the introduction of relevant policies will make CO_2_ emissions increase. From the experience of developed countries, strict environmental policy is helpful to slow down the pace of global warming. In this paper, we take the approved time of the Kyoto Protocol as a node to set the different values of the variable, and then get the conclusion that the promulgation of climate policy causes people to be eager to avoid punishment, and that carbon emissions will increase before being punished. The Kyoto Protocol requires developing countries to bear the task of reducing emissions from 2012. Because China signed the agreement in May 1998 and approved the protocol in August 2002, the responsibility for the commitment will begin from a few years later. It is possible that this will make people want to avoid or reduce punishment and then increase emissions before the arrival of the penalty period. Therefore, when formulating climate-related policies, we should not only consider the economic gap between developing and developed countries, but also solve the environmental problem from a long-term and multi-faceted perspective.

From the regression results of Model (5), Model (6), and Model (9), we can conclude that a proportion of tertiary industry is positively correlated with carbon emissions, that is, an improvement in the proportion of tertiary industry will increase the amount of carbon emissions. The reason for this result may be that producing and delivering services in tertiary industry may require a huge amount of inputs from primary and secondary industries. Hence, indirectly, the development of tertiary industry may greatly increase the carbon emissions from the manufacturing industries [46]. Low-level service industry development cannot play a role in reducing carbon emissions. In the process of industrial restructuring, although the major sectors which are responsible for carbon emissions are moving from carbon-intensive to non-carbon-intensive, more carbon emissions are generated through supply chains [47].

From the regression results of Models (5)–(8), we can conclude that ER & D investment and carbon emissions are negatively correlated. This means that technological progress in the energy industry reduces carbon emissions. Because ER & D investment directly leads to technological progress in the energy industry, this technology will also be directly applied to improve energy efficiency or new energy development projects. While the use of energy is the biggest inducement for pollutant emissions, the energy industry’s technological progress can promote the efficient use of energy, reduce pollutant emissions, and improve environmental quality. Moreover, after our special treatment of technological progress in the model, the results of the calculations also show that there is a hysteresis in the view that technological progress leads to a reduction in carbon emissions. Additionally, this hysteresis is likely to be caused by “lock-in”. Brian Arthur argues that the development process of things has a dependency on roads and rules, and it is difficult to change once the path has been chosen or the rules have been formed. This is lock-in [48]. Because early technology applications have been very mature, people have depended on old technology and been in conflict with new technology. Meanwhile, we need to take into account the increase in costs, complementary technical requirements, increased risk, and so on when talking about technological progress. These factors cause technological progress in the energy sector to not be able to achieve the expected maximum benefit to reduce maximum carbon emissions.

## 5. Conclusions

Global warming has become one of the most challenging issues about the environment. To outperform in the race of economic development, one is supposed to sacrifice the environment in exchange for the competitive advantage in the never-ending economic competition [49]. As a matter of fact, historically, most countries have prioritized the pace of their economic development and national wealth over environmental quality. After all, environmental costs are less quantitative and less measurable than the quantifiable economic indicators, such as gross domestic product. Recognizing the repercussions of global warming for the environment, a number of countries have adopted an emissions trading mechanism and a voluntary emissions reduction program to combat the global carbon emissions issue. Moreover, there are many other measures that are considered to be helpful for carbon reduction.

This paper studied whether technological progress and other factors can achieve economic development while protecting the environment from pollution based on the EKC. First of all, we confirmed that the situation in China conforms to the EKC hypothesis and the inflection point of the inverted U-shaped curve between economic growth and carbon emissions appears when the per capita GDP achieves 64,797.42 yuan. It is possible to reach the inflection point of the EKC of carbon emissions at least 5 years from now. Second, we examined the impacts of energy efficiency, climate policy, industrial structure, and an energy industry’s technological progress on carbon emissions. Among these factors, energy efficiency improvements and an energy industry’s technological progress have inhibitory effects on carbon emissions. However, the adjustment of industrial structure may increase carbon emissions. Furthermore, due to technology lock-in or other factors, the effect of an energy industry’s technological progress has a hysteresis.

Based on the above results, we can suggest the following:

1. On the road to carbon emissions reduction, China still has a lot of work to do. For example, the establishment of a carbon trading market at the end of 2017 has not been realized. The further development of renewable energy also needs to be improved. The Chinese government should strengthen regulations so that the day when economic development and carbon emissions reduction are synchronous will arrive earlier.
2. Reducing carbon emissions through improving energy efficiency is effective and should be encouraged. China’s energy efficiency has increased in recent years. For example, the unit GDP energy consumption in China was reduced by 28.6% from 2010 to 2015. However, there is still a big gap compared with developed countries. Thus, the potential benefit from energy efficiency improvement is great.
3. Energy policy development cannot just focus on a single aspect. Multi-angle considerations can make a policy more effective. For example, when adopting the Kyoto Protocol, the negative effects of time lag should be taken into account. Policy implementation should be combined with the actual market. China is implementing supply-side reform, and we expect to see effects from this reform.
4. The development of the service industry is not necessarily equivalent to the reduction of energy consumption. Because the development of tertiary industry may increase carbon emissions of other related industries, we can aim to develop a tertiary industry with limited carbon emissions, such as the financial industry [50].
5. Our results show that technological progress in the energy sector can be effective in reducing carbon emissions with a hysteresis effect. In other words, the effect of R & D investment will be shown only after some time. Due to the positive role of ER & D investment in carbon emissions reduction, government should increase it to promote technological efficiency and new technology.

There is no doubt that technological progress is a manifestation of social development. We realize that environmental pollution is serious. In order to prevent it from getting worse, we should take measures. Even if the current level of technology cannot achieve economic development and environmental protection synchronization, we should continue to work in that direction. However, one limitation of this research is that the data used is at a national level. Multi-national level data will be better because the heterogeneity between different countries can be highlighted, and policy recommendations can be more targeted. Comparing developing countries with developed countries will also be an important future research direction.

## Figures and Tables

**Table 1:** Overview of the selected studies.

Study	Estimation Method	Period	Countries	Dependent Variables	EKC Hypothesis	Linear
Caviglia Harris (2008) [15]	Two-stage least squares regression (2SLS)	1961–2000	146 countries	Ecological footprints	F	
Usama Al-mulali (2015) [16]	Generalized moment method (GMM)	1980–2008	99 countries	Ecological footprints	T in upper middle and high-income countries F in low- and lower middle-income countries	Quadratic form
Hao Yu (2014) [18]	Spatial econometric models	1995–2011	China	Energy consumption, Electricity consumption	T	Cubic form
David Katz (2015) [17]	Generalized least squares method (GLS), non-parametric regression analysis	1980–2010 1980–2005	Organisation for Economic Co-operation and Development (OECD), US	Water withdrawals, GDP	T in per capita use F in total water use	Cubic form
Álvarez Herránz (2017) [10]	GLS	1990–2014	28 OECD countries	Greenhouse gas emission	T	Cubic form
Victor Brajer (2017) [14]	OLS	1990–2004	China	SO_2_ level	T	Quadratic and cubic form
Lin Boqiang (2009) [22]	Logarithmic Mean Decomposition Method, STIRPA model	1960–2007	China	CO_2_ emission	F	
Hiroyuki Taguchi (2012) [19]	Generalized method of moments	1950–2009	19 economies in Asia	Sulphur and carbon emissions	T in sulphur emissions F in carbon emissions	Quadratic form
Thomas Jobert (2012) [31]	Iterative Bayesian shrinkage procedure, OLS	1970–2008	51 countries	CO_2_ emissions	EKC is rejected for 49 countries	Quadratic form
Khalid Ahmed (2013) [23]	Johansen cointegration Granger causality test	1980–2010	Mongolia	CO_2_ emission	T	Quadratic form
J. Wesley Burnett (2013) [25]	OLS	1981–2003	US	CO_2_ emissions	T	Quadratic form
Gu Ning (2013) [27]	OLS	1995–2009	China	CO_2_ emissions	T	Cubic form
Hu Zongyi (2013) [29]	Additive partial linear model	1980–2009	China	CO_2_ emissions	F	
Lin-Sea Lau (2014) [30]	Bounds testing, Granger causality	1970–2008	Malaysia	CO_2_ emissions	T	
Usama Al-Mulali (2016) [21]	Autoregressive distributed lag	1980–2012	Kenya	CO_2_ emission	F	Quadratic form
Kris Aaron Beck (2015) [4]	Generalized method of moments	1980–2008	OECD, Latin America Asia, Africa	CO_2_ emission	OECD countries have an N-shaped curve, Asia and Africa experience an income-based EKC pattern	Quadratic and cubic form
Miloud Lacheheb (2015) [28]	Autoregressive distributed lag	1971–2009	Algeria	CO_2_ emissions	F	
Wang Yuan (2015) [32]	Semi-parametric panel fixed effects regression	1960–2010	OECD countries	CO_2_ emissions	T	Quadratic form
Aslan Alper (2016) [24]	OLS, Granger causality test	1977–2013	China	CO_2_ emission	F	
Muhlis Can (2016) [26]	Dynamic ordinary least squares (DOLS)	1964–2011	France	CO_2_ emissions	T	Quadratic form
Wajahat Ali (2016) [20]	Autoregressive distributed lagged model, Granger causality test	1985–2012	Malaysia	CO_2_ emissions	T	Quadratic form

Notes: Only when the EKC exists, line shape will exist.

**Table 2:** Key statistics of the models.

Statics	*CO*_2_ (KG)	*GDP* (Yuan)	*EE* (Yuan/KG)	*POL*	*IND* (%)	*ZDL* (Yuan)
average	3.25	12,542.34	6.14	0.38	37.48	6.12
sd	1.64	13,818.65	4.19	0.49	6.30	9.49
min	1.52	588	0.91	0	23.2	0.05
max	6.66	47,203	15.12	1	47.8	29.97

*EE*: energy efficiency; *POL*: climate policy; *IND*: industrial structure; *ZDL*: technological progress.

**Table 3:** Comparison of Models (3) and (4).

Explanatory Variables	Model (3)	Model (4)
$GDP_{pc_{t}}$	0.173910 ***^,^^1^ (0.038909)	0.172968 *** (0.019427)
$GDP_{pc_{t}}^{2}$	−1.4 × 10^−6^ (1.83 × 10^−6^)	−1.34 × 10^−6^ *** (3.98 × 10^−7^)
$GDP_{pc_{t}}^{3}$	7.84 × 10^−13^ (2.60 × 10^−11^)	
a	1529.390 *** (237.2009)	1531.790 *** (192.7104)
Linear		inverted U
R^2^	0.992170	0.992170
AR (1) ^2^	0.634919 ***	0.634422 ***
Sample size	32	32

^1^. *** is the significance levels at 1% levels. The numbers in parentheses are standard deviations. ^2^. Autoregressive Prsocess of Order One.

**Table 4:** Comparison of Models (5)–(9).

Explanatory Variables ^1^	Model (5)	Model (6)	Model (7)	Model (8)	Model (9)
$GDP_{pc_{t}}$	0.525419 *** (0.085552)	0.281528 (0.164250)	0.347742 *** (0.055712)	0.497217 *** (0.039177)	0.659823 *** (0.070148)
$GDP_{pc_{t}}^{2}$	9.53 × 10^−6^ *** (1.4 × 10^−6^)	−1.31 × 10^−7^ (1.57 × 10^−6^)	1.2 × 10^−5^ *** (1.17 × 10^−6^)	9.56 × 10^−6^ *** (1.03 × 10^−6^)	(7.57 × 10^−6^) *** (1.03 × 10^−6^)
$EE_{t}$	−211.7729 ** (75.75468)		−47.24375 * (25.52311)	−204.4102 *** (62.74344)	−253.4086 *** (75.41654)
$EE_{t}\times GDP_{pc_{t}}$	−0.037164 *** (0.005434)		−0.046882 *** (0.004412)	−0.037354 *** (0.004229)	−0.033308 *** (0.005026)
$POL_{t}$	900.0419 ** (350.0724)	82.33858 (469.1584)		927.3813 *** (244.1749)	1272.970 *** (309.1986)
$POL_{t}\times GDP_{pc_{t}}$	0.105408	0.046070 (0.044014)		−0.107814 *** (0.033713)	−0.144701 *** (0.040652)
$IND_{t}$	11.20780 (12.74932)	0.143617 (40.11353)	12.58948 (14.16870)		21.07701 *** (7.066699)
$IND_{t}\times GDP_{pc_{t}}$	−0.000910 (0.001879)	−0.004304 (0.005042)	0.001914 (0.001689)		−0.004515 *** (0.001171)
$ZDL_{t}$	−22.36132 ** (8.695987)	−2.819548 (25.16486)	−34.58560 *** (8.051108)	−24.37024 *** (4.993454)	
a	1254.798 *** (404.4841)	1709.988 (1709.988)	1069.758 ** (444.4487)	1605.912 *** (71.47884)	945.1623 *** (186.4564)
R^2^	0.999198	0.990944	0.998890	0.999163	0.998923
Sample size	28	28	28	28	32

^1^ The first row is the parameter value. *, **, and *** are the significance levels at the 10%, 5%, and 1% levels, respectively. The numbers in parentheses are standard deviations.
